# Large and Small Yellow Croakers Feeding and Living Together Make Large Yellow Croaker Population Recovery Difficult: A Guild Perspective

**DOI:** 10.3390/biology13120963

**Published:** 2024-11-22

**Authors:** Pengyu Cai, Zhenhua Wang, Shouyu Zhang, Jintao Yu

**Affiliations:** 1 College of Oceanography and Ecological Science, Shanghai Ocean University, Shanghai 201306, China; bruinboar@gmail.com (P.C.); yujintao185061039@163.com (J.Y.); 2 Engineering Technology Research Center of Marine Ranching, Shanghai Ocean University, Shanghai 201306, China

**Keywords:** *Larimichthys crocea*, *Larimichthys polyactis*, guild, stock enhancement, overfishing, ecological niche overlap, cohabitation

## Abstract

In recent decades, China’s large-scale stock enhancement programs to restore the collapsing large yellow croaker (*Larimichthys crocea*) fishery resources have not yielded the desired results, and a comprehensive analysis of the underlying reasons for this problem is required. Based on small yellow croaker (*Larimichthys polyactis*) catch survey data obtained from 15 fishing ports along the coast of the East China Sea, we examined the proportion of large yellow croakers mixed in the small yellow croaker catch and their biological parameters. In addition, we analyzed the differences in the intestinal microbiota and feeding ecology between these two species to explore the reason why the stock enhancement program failed to achieve the desired outcome. The results show that there is a high likelihood of the two species appearing in each other’s ecological niches, and there is a significant overlap in their dietary ecology. They may cohabitate and form a guild. The fishing season targeting the small yellow croaker indirectly catches the large yellow croaker population, which puts huge fishing pressure on large yellow croaker resource and shows obvious overfishing. Therefore, it is necessary to optimize and adjust the fishing ban policy and stock enhancement strategies, appropriately reducing the fishing intensity after the fishing ban to facilitate the effective accumulation of resource replenishment effects during the fishing ban period, thus effectively restoring wild large yellow croaker resources.

## Simple Summary

Due to unrestrained and excessive fishing, the migratory fishing season and spawning groups of the large yellow croaker (*Larimichthys crocea*) in the East China Sea have been hard to detect since the 1980s. Since the 1990s, the Chinese government has initiated large-scale stock enhancement programs to restore the endangered wild large yellow croaker population. However, after several decades, the stock enhancement has not achieved its desired effects. The reasons for this remain poorly understood. Our results provide evidence that in the early-growth stage of the large yellow croaker, when its body length and body mass are similar to those of the small yellow croaker (*Larimichthys polyactis*), it may have a life history strategy of cohabitation and forming a guild with the small yellow croaker. Since the fishery resources of the small yellow croaker are far higher than those of the large yellow croaker, fishing targeting the small yellow croaker will indirectly lead to the large-scale capture of the cohabitate large yellow croaker, dealing a heavy blow to the slightly recovering fishery resources of the large yellow croaker. Therefore, optimizations and adjustments are necessary in order for the fishing ban policy and stock enhancement strategy to effectively restore the resources of the large yellow croaker.

## 1. Introduction

The excessive exploitation of marine resources combined with the intensive discharge of wastewater significantly impairs the capacity of the primary elements in the aquatic ecosystem to be transformed and utilized by higher trophic-level organisms [1,2]. Due to the scarcity of adequate predators, significant energy accumulates in small or juvenile organisms, thereby resulting in a higher local abundance of prey organisms [3]. In this context, some already highly differentiated biological populations will inevitably exhibit cohabitation due to insufficient competitive conditions, which ultimately leads to abundant guilds in the early stages or specific phases of their life history [4,5].

In ecology, a guild is a collective of species that utilize similar types of resources in analogous ways. These groups frequently consist of closely related species with a shared ancestor; thus, they exploit resources in comparable ways due to their common lineage. Consequently, various species within a particular genus may form a guild within a community [6,7]. One advantage of guild studies is the simplification of community analysis by providing an operational unit that bridges the gap between individual species and the entire community. This aids the streamlining of data collection under constraints on funding or personnel [6] and allows for the inference of dynamic changes in other guild members based on monitoring a single member within the guild and tracking fluctuations in its abundance [8]. Therefore, research into guilds can contribute to conservation efforts for severely threatened biological populations. The concept of a guild was originally proposed for analyzing avian communities but has since been further applied to studies of fish communities [9,10]. Previous studies of guilds primarily focused on habitat utilization [11,12], guild structure [13,14], and co-occurrence guild food web dynamics [15,16]. However, comprehensive systematic studies of the impacts of economic marine fish guilds on resource utilization and conservation are still relatively scarce.

The large yellow croaker (*Larimichthys crocea* (Richardson, 1846)) and the small yellow croaker (*Larimichthys polyactis* (Bleeker, 1877)) both belong to the family Sciaenidae. They are important economic demersal species in the East China Sea. During the overwintering grounds, large yellow croakers and small yellow croakers inhabit different water layers in the offshore regions, but as they migrate towards the coastal areas, they share the same feeding and spawning grounds, leading to habitat overlap [17,18]. The appearance and coloration during the early-life stages, not yet having reached initial sexual maturity, are similar for the large yellow croaker and small yellow croaker, so it is difficult for fishermen to distinguish them based on naked-eye observations [19]. Previous regional fisheries resource surveys in the fishing boats moored at the same fishing port by our team observed that fishermen often mistakenly identified young large yellow croakers as small yellow croakers or labeled them as “small-scaled small yellow croaker” for sale [20]. Consequently, significant proportions of large yellow croaker were mixed with the small yellow croaker catch on the market. A limited number of previous studies also reported similar findings, albeit without thoroughly elucidating the underlying scientific issues [21]. The large yellow croaker is one of the four traditional major economic marine fish species in China, alongside the *Larimichthys polyactis*, *Trichiurus japonicus*, and *Sepiella japonica*. It has a pivotal position in the marine fish fauna structure of China and has become intertwined with the folk culture of the coastal areas of the East China Sea. After the 1950s, the fishing pressure on wild large yellow croakers steadily increased. Following a peak of around 200,000 tons in the mid-1970s, the catch plummeted by over 90% within a mere 20 years. Subsequently, wild large yellow croaker resources declined sharply, leading to its classification as critically endangered (CR) by the International Union for Conservation of Nature (IUCN 2016 ver. 3.1) [22]. Since a breakthrough in the breeding technology for large yellow croakers in the 1980s, the provinces of Fujian, Zhejiang, and Jiangsu initiated stock enhancement with juvenile breeding large yellow croakers in coastal waters to supplement the natural population and improve the aquatic ecosystem [21]. Subsequently, the scale and frequency of stock enhancement for large yellow croakers gradually increased in the hope of restoring wild large yellow croaker resources. According to the data, from 1987 to 2018, Fujian Province released a total of 115 million large yellow croakers [23]. During the 5 years from 2017 to 2021, Zhejiang Province released a total of 303,198,000 large yellow croakers, with an average release size of 6.5 cm and a total investment of 64.049 million yuan [24]. Overall, the enhancement of the large yellow croaker stocks in China is now conducted on a massive scale, and the stock enhancement activities are mainly conducted during the fishing ban period with the aim of achieving better outcomes.

Despite the massive scale and frequency of release, only a limited number of studies have indicated that supplementation of the natural large yellow croaker population by stock enhancement has been effective [25]. However, the reported catch of wild large yellow croakers in the main release regions in China (Zhejiang, Fujian, and Jiangsu) has not to show significant growth [26]. The important spawning grounds in the East China Sea still fail to generate a fishing season, and there are no clear signs of recovery in the wild large yellow croaker fishery resources [23]. Some studies even suggest that China’s large yellow croaker stock enhancement programs over the past few decades have not to be successful [22]. However, these studies only provided limited insights into the role of stock enhancement in the recovery of wild resources of the large yellow croakers, and they failed to elucidate the reasons behind the prolonged lack of recovery in large yellow croaker fishery resources and fishing seasons. We speculate that against the backdrop of large-scale stock enhancement, large yellow croakers in the early-life stages may have a profound cohabitation with small yellow croaker populations and form a typical guild. The small yellow croaker is one of China’s traditional four major economic marine fish species, and production is relatively high in China. Furthermore, based on the China Fishery Statistical Yearbook, the data indicate that the output of the small yellow croaker along the coast of the East China Sea in 2022 was 268,730 tons, while that of the large yellow croaker was merely 37,098 tons, with the former being seven times that of the latter. If a large-scale cohabitation phenomenon exists between the large yellow croaker and the small yellow croaker, it is likely that the reasonable fishing of the small yellow croaker may, unintentionally, deal a heavy blow to the fishery resources of the large yellow croaker. Therefore, understanding the mechanisms and intensity of this cohabitation between the two species is of great importance for the conservation of the large yellow croaker. Moreover, in the future, taking the guild as the unit will enable more scientific and rational promotion of the assessment and protection of the fishery resources of the large yellow croaker, as well as the management of its stock enhancement. Therefore, in this study, we conducted a field investigation based on the catches of fishing boats at fishing ports in three provinces of China’s nearshore waters to obtain insights into the actual scale of fishing for large yellow croakers to infer the reasons for the lack of success in stock enhancement efforts in China over the years. We also compared the intestinal microbial structures and feeding ecology of the large yellow croaker and the small yellow croaker and aimed to verify whether the large yellow croaker and the small yellow croaker are in cohabitation and whether they form a guild. By better understanding the resource structure during the transition from young to adults of the large yellow croaker, the results obtained in this study can inform the management of large yellow croaker fishery resources and subsequent stock enhancement efforts.

## 2. Materials and Methods

### 2.1. Sample Collection and Morphological Identification Methods

**Figure 1:**
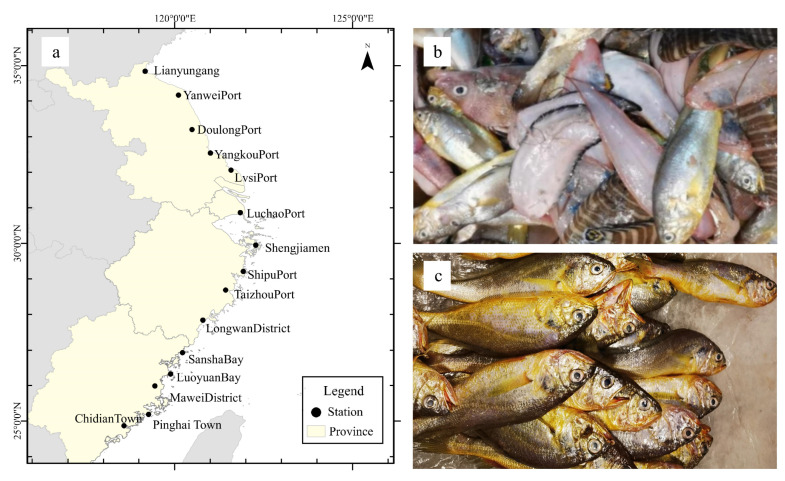
(**a**) Distribution map of coastal survey sampling sites. (**b**) One of the catches from the trawler boat. (**c**) Fishermen will mix the large yellow croaker and the small yellow croaker for sale after preliminarily sorting their catch.

During our team’s past investigations of fishery resources in local sea areas off the coast of Zhejiang and the catches of fishing boats at Luchao Port in Shanghai, it was observed that from 2018 to 2022, during the autumn months following the fishing ban period from May to September, large and small yellow croakers smaller than 20 cm were frequently captured together, while a similar phenomenon rarely occurred in the following spring [20]. To verify whether this phenomenon exists universally in the East China Sea and to eliminate the intense interference of fishing activities and obtain the catch information that best represents the actual situation, this study was carried out during the first large-scale trading cycle after the closed fishing period in the autumn of 2023. The sampling period was from late September to early October. Considering the distribution characteristics of coastal fishing port cities and the four major sea areas from south to north in China, a total of 15 sampling sites were designated across three provinces (Figure 1). At each station, the catches of yellow croakers from three different fishing boats were randomly collected and identified. To ensure the reliability of sample sources, information on the fishing area should be obtained from fishermen before the identification of the catch to make sure the samples are from the local sea area and inquire about the approximate fishing area range. Then, morphological identification is carried out on the fish catch to distinguish large yellow croakers and small yellow croakers, and the number and proportion of the two different catches at different stations are recorded. In this study, a total of 8330 yellow croaker samples were classified and identified. For each sampling site, the total length (TL) and body mass of both the large yellow croaker and the small yellow croaker were measured on the spot, with a total of 883 individual measurements taken for each species. Body length was measured using a standard ruler (to the nearest 1 mm), and body mass was measured using an electronic balance (to the nearest 0.01 g). Additionally, 30 large yellow croakers were randomly sampled from the catch of every sampling site, and when the number of the large yellow croakers caught was less than 30, all were sampled, along with an equal number of the small yellow croakers. A total of 402 samples of both the large yellow croaker and the small yellow croaker were collected, respectively. All samples were transported back to the laboratory under low-temperature preservation conditions for subsequent experiments. The catches were primarily obtained from trawl nets and gill nets. We obtained the fishing boat number and obtained the AIS data through the Hifleet website (https://www.hifleet.com (accessed on 27 December 2023)) to receive its fishing trajectory and draw the contour of the working area of different boats.

**Figure 2:**
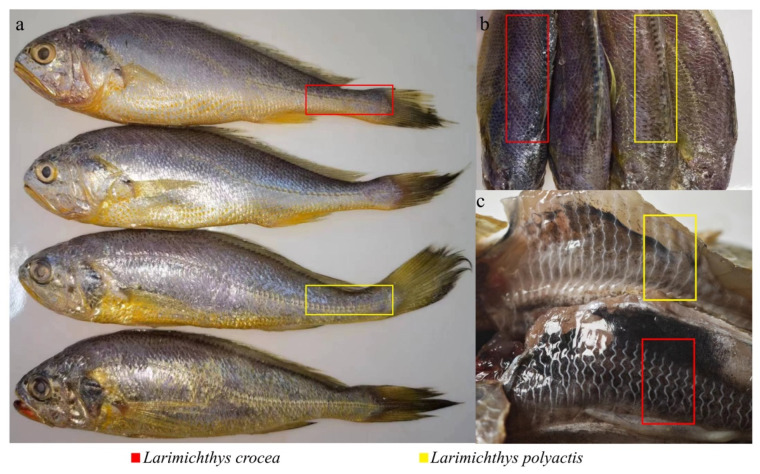
The morphological distinctions between large and small yellow croakers [19], (**a**,**b**) depict the varying quantities of scales in two fish species, while (**c**) illustrates the differences in the swim bladder lateral branchs of the two fish species.

The differentiation between large yellow croakers and small yellow croakers primarily relies on morphological identification methods. Small yellow croakers exhibit 5–6 rows of sparse scales from the dorsal fin origin to the lateral line, whereas large yellow croakers display 8–9 rows of densely packed scales (Figure 2b). In terms of lateral line characteristics, small yellow croakers have a thick midline pattern with a gentle mid-body concavity, while large yellow croakers feature a fine midline pattern with a sudden mid-body concavity (Figure 2a). The collected samples were further species-confirmed in the laboratory through the examination of the swim bladder lateral branches. In small yellow croakers, the two small branches at the margin of the branch of the swim bladder lateral branch are of unequal length, whereas in large yellow croakers, these two small branches are of equal length (Figure 2c).

### 2.2. Stable Isotope Analysis Methodology

Following the conclusion of biological indicator measurements, a total of 40 specimens of large and small yellow croakers, 10 from each, were selected randomly from the regions of Zhoushan and Taizhou. The fish were meticulously cleansed of impurities using ultra-pure water. Subsequently, rectangular muscle tissue measuring 2 cm^2^ from below the dorsal fin was carefully excised and subjected to a 24 h drying process in the freeze dryer (Alpha 1-2LDplus, Martin Christ, Osterode, Germany) before being ground into powder for further analysis. A sample weighing 1.5 mg was meticulously encapsulated in tin foil and subjected to analysis for carbon and nitrogen stable isotope ratios using the DELTA V Advantage Isotope Ratio Mass Spectrometer in conjunction with Thermo Fisher Scientific’s EA-HT Elemental Analyzer (Waltham, MA, USA). The stable carbon and nitrogen isotope values were determined relative to the internationally recognized standards: Pee Dee Belemnite (PDB) for carbon and atmospheric nitrogen for nitrogen. The results are expressed in the form of a carbon stable isotope ratio (δ ^13^C) and a nitrogen stable isotope ratio (δ ^15^N). The calculations for δ ^13^C and δ ^15^N were performed using the following formula:

$$\delta \;X=\left[R_{sa}/R_{st}-1\right]\times 1000.$$

In the formula provided: X represents either ^13^C or ^15^N; and *R* denotes the ratio of ^13^C/^12^C or ^15^N/^14^N between the sample under analysis (*R_sa_*) and the standard reference (*R_st_*). The analytical precision achieved is 0.1‰ for δ ^13^C and 0.06‰ for δ ^15^N.

Under the assumptions of normal distribution and homogeneity of variance in the data, a one-way analysis of variance (ANOVA) was conducted to test for significant differences in δ ^13^C and δ ^15^N values between the two fish species at a significance level of α = 0.05. Nutritional levels were calculated based on the formula as described by Vander [27].

$$TL=\left[\left(\delta^{15}N_{c}-\delta^{15}N_{b}\right)/TEF+\lambda \right]$$

In the formula provided: TL represents the trophic level estimated for the organism; δ ^15^*N_c_* denotes the δ ^15^N ratio of the consumer; while δ ^15^*N_b_* represents the δ ^15^N ratio of the baseline organism. For this study, the baseline organism selected was the thick-shelled mussel (with a δ ^15^N ratio of 3.9‰), a year-round presence in the Zhoushan Sea area with a simple diet. *TEF* stands for the trophic enrichment factor for a nutritional level, set at 3.4‰ in this context. λ represents the trophic level of the chosen reference organism, which is 2 in this case.

### 2.3. High-Throughput Sequencing of Genetic Material in Gastric Contents

Ten large yellow croakers collected randomly from the Zhoushan Sea area were dissected using ethanol-disinfected dissecting scissors. The stomach pouches were opened along the gastric wall, and all gastric contents were scraped off. Gastric contents of the same species were grouped, crushed, and quantified. Subsequently, DNA extraction was performed using the QIAamp Power Fecal Pro DNA Extraction Kit (Tianlin Biotechnology Co., Ltd., Shanghai, China)) according to the manufacturer’s instructions. The obtained DNA was subjected to 1% agarose gel electrophoresis for detection, quantified, and then stored. Universal primers mlCOIintF (5′-GGWACWGGWTGAACWGTWTAYCCYCC-3′) and jgHCO2198 (5′-TAIACYTCIGGRTGICCRAARAAYCA-3′) were utilized for amplification [28]. The mitochondrial cytochrome c oxidase subunit I (COI) gene was amplified via PCR, yielding a gene fragment of approximately 313 bp in length. The PCR reaction mixture, totaling 25 uL, consisted of 12.5 uL Taq PCR Master Mix, 9.5 uL ddH2O, 1 uL each of forward and reverse primers, and 1 uL of DNA template. The reaction program was set as follows: initial denaturation at 94 °C for 4 min, denaturation at 94 °C for 30 s, annealing at 55 °C for 30 s, extension at 72 °C for 45 s, followed by a final extension at 72 °C for 10 min, with a total of 30 cycles. After amplification, the products were subjected to 2% agarose gel electrophoresis for verification and then underwent paired-end sequencing on the Illumina Miseq platform (Shanghai Paisenno Biotechnology Co., Ltd., Shanghai, China).

In Excel (Microsoft Excel 2023), the relative abundance of OTUs at the phylum level was statistically analyzed. Simultaneously, the OTU species with the highest relative abundance were selected for Blast alignment to explore information on closely related species. Following the removal of sequences related to large and small yellow croaker, OTU sequences with a minimum of 90% sequence similarity were identified based on the analysis of their gastric contents and the aquatic biota catalog of the East China Sea. Subsequently, using MEGA11 (Tamura, Stecher, and Kumar 2021), the neighbor-joining algorithm and maximum likelihood estimation were employed to construct a phylogenetic tree of the bait organisms, with a bootstrap value set to 1000 iterations. Additionally, based on the relative abundance of OTUs from the bait organisms, a heatmap analysis was conducted in Excel to investigate feeding differences between the two fish species.

### 2.4. Sample Grouping and Extraction of Intestinal DNA for High-Throughput Sequencing Analysis

Sixteen yellow croakers were randomly selected, including four large yellow croakers (LZS) and four small yellow croakers (SZS) from Zhoushan, as well as four large yellow croakers (LTZ) and four small yellow croakers (STZ) from Taizhou. The large and small yellow croakers were delicately wiped with 75% alcohol and underwent aseptic dissection to extract the digestive tract, and the contents of the rectums from each fish were meticulously collected, blended, and preserved by flash freezing them in liquid nitrogen. The extraction of intestinal microbial DNA was conducted using the E.Z.N.A™ Mag-Bind Soil DNA Kit (Guangzhou, China), following a detailed procedure as outlined in the product manual. Post extraction, the DNA underwent assessment for concentration and purity before being subjected to PCR amplification targeting the V3-V4 hypervariable region of the 16S rDNA sequence. The primers used for amplification were 338F (5′-ACTCCTACGGGAGGCAGCA-3′) and 806R (5′-GGACTACHVGGGTWTCTAAT-3′). Upon successful validation of the amplification products through agarose gel electrophoresis, a library was constructed, and high-throughput sequencing was carried out on the Illumina MiSeq PE300 platform. The specific operational steps followed the methodology outlined by Zhang [29].

The paired-end reads obtained from intestinal microbiota sequencing were concatenated, filtered, and dechimerized to generate the final clean data in fastq format. The Qiime 1.9.1 standardized pipeline was employed, utilizing the open reference algorithm to cluster OUTs at 97% similarity, followed by alignment with the GreenGene database to create a Biom dataset containing sample names, taxonomic annotations, and abundance information. Alpha diversity indices (OTU, Chao1, PD whole tree, Shannon, and Simpson indices) of the samples were analyzed using Mothur 1.30 software, while Beta diversity (Bray Curtis PCoA and Weighted Unifrac PCoA) analysis was conducted using QIIME software (version 1.8.0). The species abundance of the samples was then examined at the phylum and genus levels to analyze the composition of the microbial community.

## 3. Results

### 3.1. Proportions of Large Yellow Croaker Catches to Small Yellow Croaker Catches at Different Sampling Sites

The proportions of the large yellow croakers were determined at different sampling sites (Table 1), and the results showed that the proportion of the large yellow croakers at the 15 sampling sites ranged from 5.45% to 34.08%, with the lowest proportion at Yanwei Port (5.45%) and the highest at Taizhou Port (34.08%). The proportion of the large yellow croaker in the small yellow croaker samples was highest in Zhejiang (25.91 ± 7.53%), followed by Fujian (24.69 ± 8.78%), and lowest in Jiangsu (15.77 ± 9.15%). The average proportion of the large yellow croaker in the small yellow croaker catch in China’s nearshore waters was 21.44 ± 9.35%.

**Figure 3:**
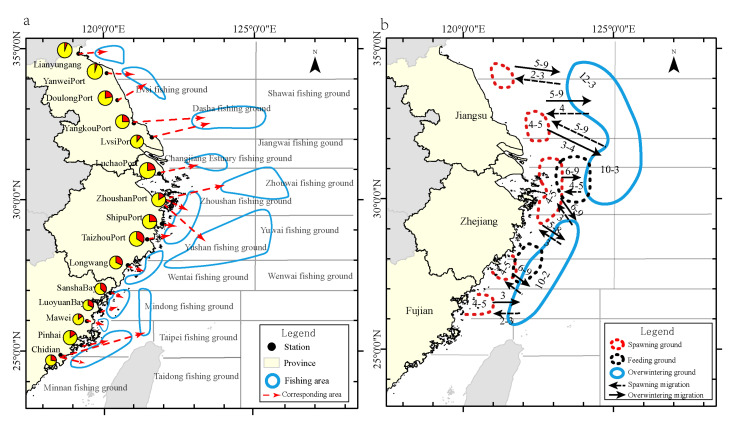
Proportions of large and small yellow croakers in fish catches at different survey stations and fishing areas: red color represents the large yellow croaker; yellow color represents the small yellow croaker (**a**). Historical spawning ground, nursing ground, overwintering ground, and migration route for the large yellow croaker. The numbers on (**b**) indicate the months when large yellow croakers migrate [30].

The different fishing areas and historical large yellow croaker migration routes are shown in Figure 3a. The main catches of the large yellow croaker fisheries come from Lvsi Fishing Ground, Zhoushan Fishing Ground, Changjiangkou Fishing Ground, Zhouwai Fishing Ground, and Minzhong and Mindong Fishing Ground. As shown in Figure 3b, the current large yellow croaker fishing area overlaps with part of the historical spawning ground, nursing ground, overwintering ground, and migration route of the large yellow croaker in the East China Sea.

### 3.2. Body Length and Body Mass Composition of the Large Yellow Croaker

**Figure 4:**
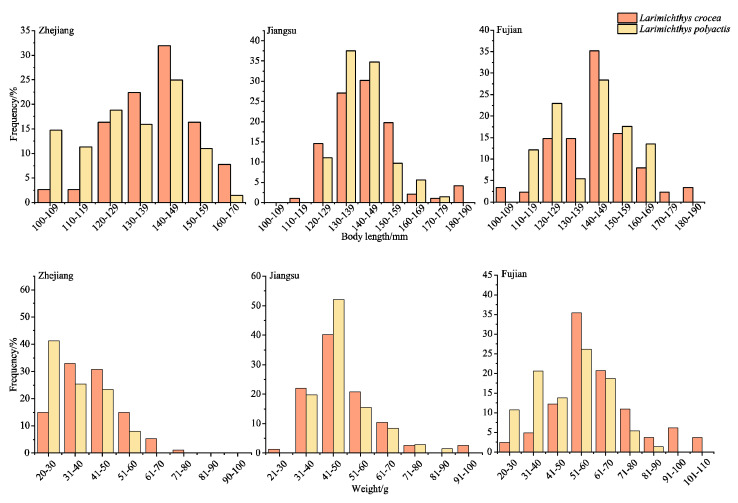
Frequency distribution of body length and body mass for large and small yellow croakers in different regions.

The body length range in the large yellow croaker samples obtained from the coastal survey was 99 to 187 mm, with an average body length of 141.73 ± 14.61 mm, and the dominant body length group was 125 to 160 mm, accounting for 87.6% of the total. The average body length of the large yellow croaker collected in Jiangsu was the largest, followed by Fujian and Zhejiang. The body mass ranged from 12.7 to 135.08 g, with an average body mass of 49.45 ± 17.77 g, and the dominant body mass group was 30 to 75 g, accounting for 82.2% of the total (Table 2) (Figure 4). It can be determined from Table 2 that there is no significant difference in body length and body mass between the large yellow croaker and the small yellow croaker as collected in this study (*p* > 0.05). Accordingly, we can infer that in the early-life stage of the large yellow croaker, when its body length and body mass are similar to those of the small yellow croaker, it may be more likely to have a life history strategy of cohabiting with small yellow croaker.

### 3.3. Sex Ratio and Gonadal Maturity of the Large Yellow Croaker

**Figure 5:**
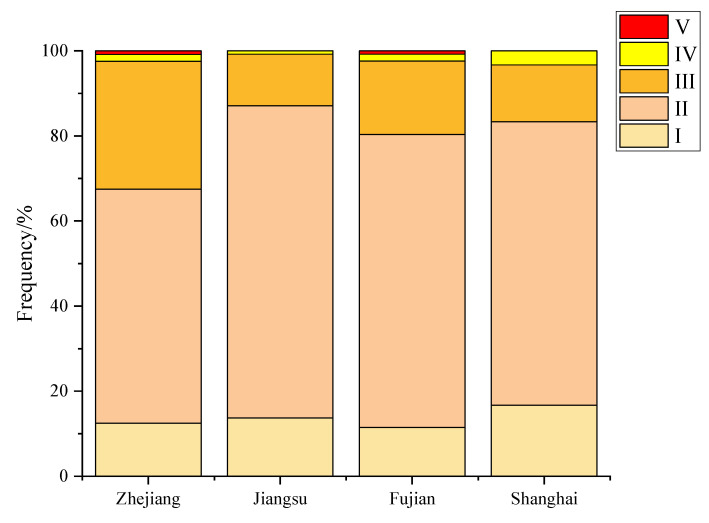
Proportions of large yellow croakers in different gonadal maturity stages.

As shown in Figure 5, the gonadal maturity of the large yellow croaker caught in China’s nearshore waters was mainly at stage II, accounting for 63.43%, with 20.39% at stage III, 1.4% at stage IV, 0.4% in the spawning state (stage V), and 0% at stage VI. Thus, gonad development was premature in some large yellow croakers, which was related to the ecological adaptation of the large yellow croaker under the background of transition fishing.

### 3.4. Stable Isotope Characteristics and Interspecific Nutritional Hierarchy Differences

The δ ^13^C and δ ^15^N values for the large yellow croaker and the small yellow croaker are shown in Table 3. The total δ ^13^C values for the small yellow croaker ranged from −18.64‰ to −15.30‰, with a maximum difference of 3.34‰. The δ ^13^C values for the large yellow croaker ranged from −17.22‰ to −15.88‰, with a maximum difference of 1.34‰. The mean value was −19.62 ± 1.11‰, and the small yellow croaker had a higher mean δ ^13^C value than the large yellow croaker. The total δ ^15^N ratios for the small yellow croaker ranged from 9.95‰ to 12.04‰, with a maximum difference of 2.09‰ and a mean value of 10.71 ± 0.50‰. Small yellow croakers had a higher mean value compared with large yellow croakers. Univariate analysis of variance indicated no significant differences in the carbon and nitrogen stable isotope ratios between large and small yellow croakers (*p* > 0.05).

By using *Mytilus coruscus* (δ^15^N value of 3.9 ‰) as the reference organism, the average trophic levels of large and small yellow croakers in Zhoushan and Taizhou waters were calculated based on the stable isotope ratio analysis results as 4.16 ± 0.05 and 4.15 ± 0.03, respectively. One-way analysis of variance showed that there were no significant differences in the trophic levels between large and small yellow croakers in different regions (*p* > 0.05).

**Figure 6:**
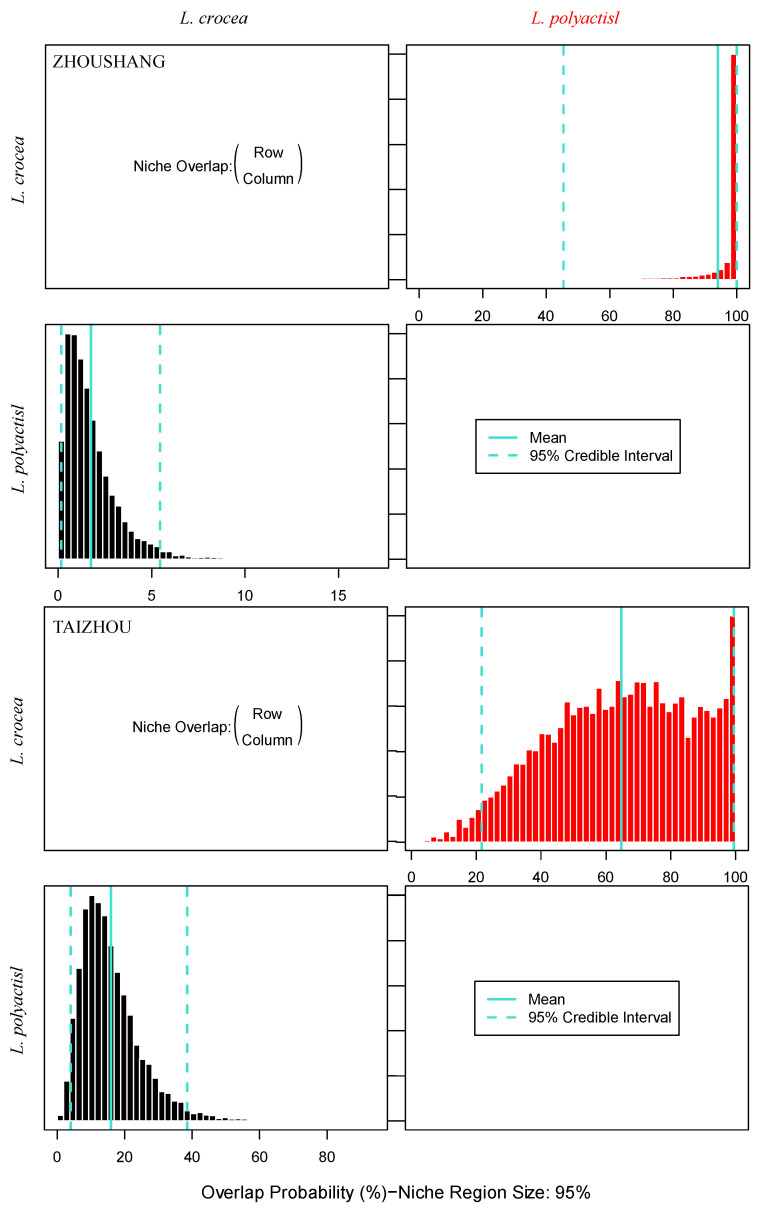
Bayesian plot of the posterior probability distributions of species niche regionalization measures (%) obtained using nicheROVER, with the posterior mean and 95% credible interval displayed in blue.

Using the nicheROVER niche model, the degree of niche overlap between the two species was calculated. The results showed that the nutrient niches of the two species overlapped. Figure 6 shows the posterior distribution of the niche overlap measure. nicheROVER analysis indicates the probability that a species is found in the niche region of another species. In the Taizhou and Zhoushan regions, the probabilities that the stable isotope niche of the large yellow croaker overlapped with the niche region of the small yellow croaker were 65% and 94%, respectively.

### 3.5. Comparison of Gastric Contents of the Two Fish Species

**Figure 7:**
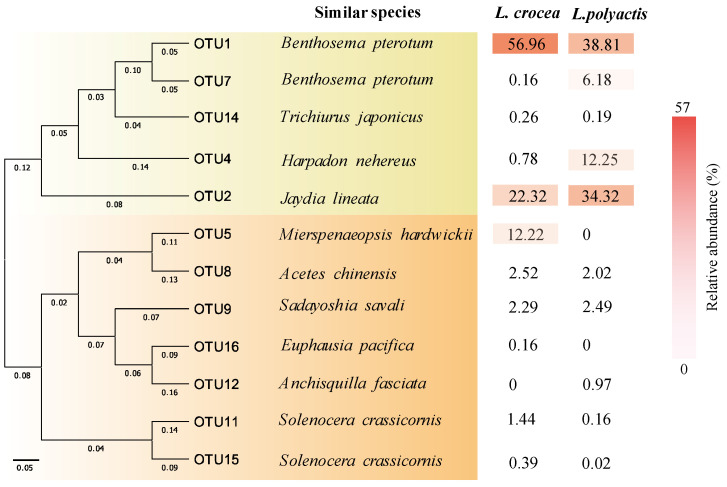
High-throughput sequencing analysis of COI gene to assess the dietary compositions for the large yellow croaker and the small yellow croaker.

Analysis of the gastric contents of the two fish species is shown in Figure 7. We find that in the large yellow croaker, the dominant operational taxonomic unit (OTU) in its food was OTU1 (relative abundance of 56.96%), which was similar to *Benthosema pterotum*, and the second was OTU2 (relative abundance of 22.32%), which was similar to *Jaydia lineata*. The relative abundance of OTU5 was also relatively high (there was a relative abundance of 12.22%), and it was similar to *Mierspenaeopsis hardwickii*. The dominant OTUs in the diet of the small yellow croaker were similar to those for the large yellow croaker, where the main food items were *Benthosema pterotum* and *Jaydia lineata*, with a relative abundance of 38.81% and 34.32%. The relative abundance of OTU4 was also relatively high (there was a relative abundance of 12.25%), and it was similar to *Harpadon nehereus*. The relative abundance values for all other OTUs in the stomach contents were less than 3.0%.

### 3.6. Analysis of Intestinal Microbial Diversity

**Figure 8:**
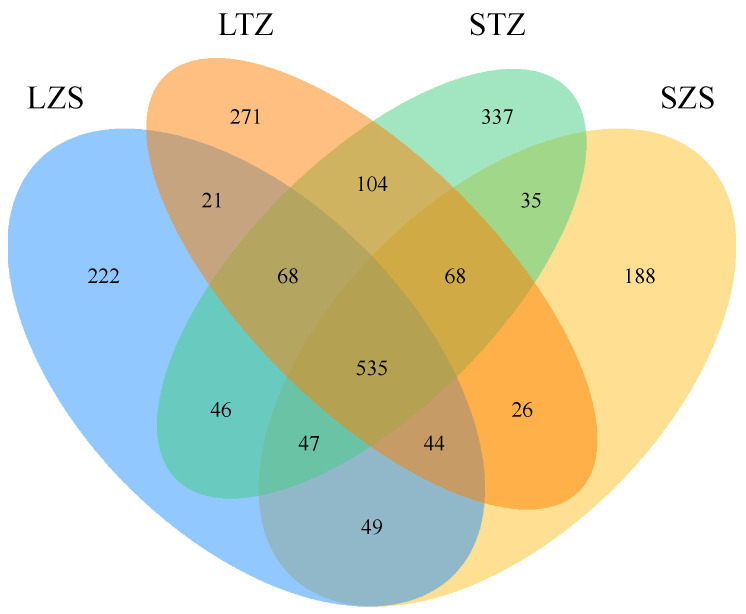
Venn diagram showing the intestinal microbiota in large yellow croaker and small yellow croaker in different regions.

Figure 8 presents a Venn diagram illustrating the OTU feature sequences for large yellow croakers (LZS) and small yellow croakers (SZS) collected in Zhoushan, as well as those for large yellow croakers (LTZ) and small yellow croakers (STZ) collected in Taizhou. The total number of OTUs shared between the two marine regions for both species was 535, representing 25.9% of the overall total. The large and small yellow croakers in the Zhoushan region had 357 and 317 unique OTUs, respectively, with a combined total of 675 OTUs, constituting 50.03% of the total for both species. In the Taizhou region, the large and small yellow croakers together had 1147 OTUs, representing 59.4% of the total for both species. Clearly, the intestinal microbiota compositions were similar for large and small yellow croakers in the two marine regions.

The α-diversity indices obtained for intestinal microbiota can directly reflect the species diversity within a group. To investigate differences in the richness and diversity of bacterial communities in intestinal samples, α-diversity analysis was conducted based on sequencing data. The results indicated that there were no significant differences in the Shannon, Simpson, Chao1, and Ace indices between large and small yellow croakers from the same region (Table 4) (*p* > 0.05).

**Figure 9:**
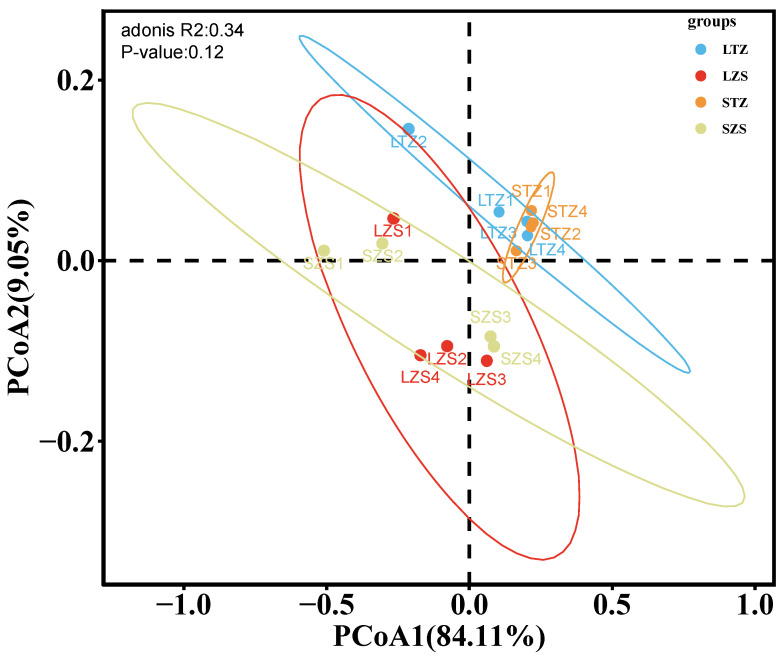
Principal coordinates analysis (PCoA) results based on weighted Unifrac distances. Note: Each point in the figure represents one sample, samples from the same group are depicted in the same color, and the color regions indicate confidence intervals. LZS 1–4 represent the four samples from the large yellow croaker group in Zhoushan; SZS 1–4 represent the four samples from the small yellow croaker group in Zhoushan; LTZ 1–4 represent the four samples from the large yellow croaker group in Taizhou; STZ 1–4 represent the four samples from the small yellow croaker group in Taizhou. The same applies to Figure 10.

Principal coordinates analysis (PCoA) was performed based on weighted Unifrac distances to assess the β-diversity of different groupings. The PCoA plot (Figure 9) showed that there were no significant differences between large and small yellow croakers in the Taizhou marine area (PerMANOVA) (*p* = 0.77), and no significant difference between the two species in the Zhoushan marine area (*p* = 0.82). These results indicate that large and small yellow croakers had similar characteristic intestinal microbiota communities in both marine areas.

### 3.7. Comparison of Intestinal Microbiota Structure

**Figure 10:**
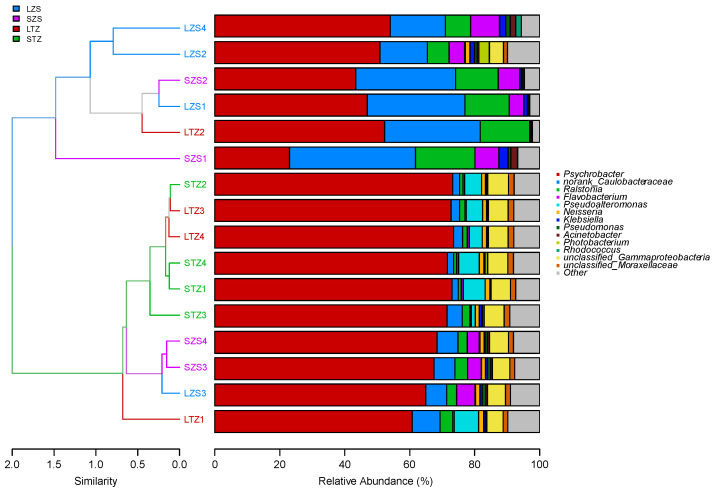
Sample cluster tree and histogram combined analysis diagram.

The left-hand side of Figure 10 shows a dendrogram representing the similarity between samples, where samples with minor differences are clustered together on the same branch and distinguished by color based on group information. The right-hand side shows a bar graph illustrating the species distribution within each sample. In the dendrogram, the large yellow croaker group and small yellow croaker group are situated on the same branch, thereby indicating a lack of significant differences between the two sample sets. The relative abundance plot (Figure 10) shows that *Psychrobacter* was the dominant genus for both large yellow croaker and small yellow croaker samples. In total, 530 genera were identified in the large yellow croaker and small yellow croaker samples, and the compositions of the dominant genera were similar in each sample, where they consisted of *Psychrobacter*, *Caulobacteraceae*, *Ralstonia*, and *Flavobacterium*. Overall, except for *Pseudoalteromonas* and *Neisseria*, the compositions of the other dominant bacterial genera were similar, and the similarity of the intestinal microbial composition was high at the genus level in large yellow croaker and small yellow croaker.

## 4. Discussion

### 4.1. Stock Enhancement During the Fishing Ban Significantly Affected the Catch Ratios of Two Sciaenidae Species

Under the background that the fishery resources of the large yellow croaker in the East China Sea of China remain depressed at present, and the migratory fishing seasons and spawning groups are still hard to find, we consider that the vast majority of the large yellow croakers which were caught and marketed as small yellow croakers are mostly from stock enhancement during the fishing ban. Our results showed that as a result of large-scale stocking activities in the coastal areas of three provinces in the East China Sea after the fishing ban period, the average catch proportion of the large yellow croaker in the three provinces accounted for 21.44 ± 9.35% of the total yellow croaker catch. Compared with the average release size of 65 mm, the average length of these catches was 147.3 mm. Based on the Von Bertalanffy growth model ($L_{t}=481\times \left(1-e^{-0.5\times \left(t+0.25\right)}\right)$) obtained from previous studies on the expected growth pattern of the large yellow croaker in the East China Sea [31], it can be calculated that the theoretical body length of the large yellow croaker released for stock enhancement at the beginning of the fishing ban in May of the current year would be 142.05 mm after five and a half months of growth, which is consistent with the aforesaid results. This result indicates that large yellow croaker juveniles released in the year could feed and grow under natural conditions. Meanwhile, studies have indicated that there is no significant difference in body length and body mass between the large yellow croaker and the small yellow croaker, which, together, constitute the guild. Accordingly, we can speculate that in the early-life stage of the large yellow croaker, when its body length and body mass are similar to those of the small yellow croaker, it is more likely to have a life history strategy of cohabitating with them. According to the fishing production data reported in the China Fishery Statistical Yearbook (2023) [26], China’s catch volume of the small yellow croaker in 2022 was 268,000 tons, whereas the catch volume of the large yellow croaker was only 37,000 tons. Based on the results, we can infer that the actual catch volume of the large yellow croaker in that year was significantly higher than the reported figure, thereby highlighting the positive impact of stocking on resource recovery. However, during a repeated survey of the landing catches at LuChao Port in Shanghai from March to April in the following year, that is, half a year after the end of the stock enhancement, it was discovered that the proportion of large yellow croaker catches decreased to less than 3%; thus, there was a sharp decline in the recently recovering large yellow croaker resource abundance. In addition, the fishing production data reported in the China Fishery Statistical Yearbook (2023) indicate that the catch volume of the large yellow croaker in 2022 decreased by 10,600 tons compared with 2021, i.e., a decrease of 2.8%. In particular, the catch volume of the large yellow croaker of Guangdong and Hainan provinces in the South China Sea reached 29,000 tons, accounting for 79.4% of the national catch volume. Thus, the enhancement of the large yellow croaker stocks in the coastal provinces of eastern China did not increase its fishable resource abundance, which our results suggest was due to the large yellow croaker released for stocking during the closed fishing period being caught along with the small yellow croaker after the fishing ban was lifted, and this led to underestimation of the yield of the large yellow croaker and had a detrimental effect on the initially successful stocking programs. However, there have been occasional news reports in recent years of large-sized large yellow croakers weighing over 1 kg being caught in the East China Sea [32,33]. Moreover, large yellow croaker populations have even been discovered in the Ma’an Islands, thereby highlighting the ability of the released but uncaught large yellow croaker to thrive in habitats such as island reefs and to contribute to the restoration of wild populations. Meanwhile, through historical fisheries statistics and estimations from other researchers on the East China Sea large yellow croaker fishery resources [30], we have found that compared to the near-extinct population of the large yellow croakers in the 1990s, the current population has recovered. This recovery can be attributed to the working of the restocking program. Therefore, we consider that enhancing large yellow croaker stocks is important for resource recovery.

### 4.2. Guild of Large and Small Yellow Croakers Influences the Conservation of the Large Yellow Croaker Species

Our investigations of intestinal microbiota, stable isotopes in muscle tissues, and stomach content compositions showed that the early-life stages of the large yellow croaker released in the sea areas of Zhejiang formed a typical guild with small yellow croakers, with cohabitation in the same habitat and utilization of similar resources. Previous studies have shown that the current environmental conditions in the East China Sea can provide sufficient and rich food resources for both large yellow croaker and small yellow croaker fish species, and there is no intense interspecies competition. For example, Xu [3] found that the current summer fishing ban system has promoted structural improvement of the East China Sea ecosystem and the recovery of food resources. Li [34] found that the food source diversity level and nutritional trophic level span were relatively high and stable in the feeding ground of the East China Sea southwestern waters. The current habitat provides sufficient food and good ecosystem structure, which alleviates resource competition and promotes the formation of the guild. We believe that in the policy context of the long-term implementation of the summer fishing ban, the absence of intense competition will be sustainable in the long term. However, it should be noted that the current summer fishing ban system has only achieved short-term effects on the utilization of resources, with an increase in resources for a short period of time. After the end of the fishing ban period, massive amounts of the guild of large and small yellow croakers were caught. This strong fishing effort for the small yellow croaker in a short period negated the restoration effects of the fishing ban system, indirectly hindering the recovery of large yellow croaker resources and preventing the sustainable effects of stock enhancement. In studying the impact of the fishing ban on fishery resources, Barnes et al. [35] established a closed-area model to determine the impact of the fishing ban on fisheries production. Their research revealed that the recovery time of fish resources is correlated with the fishing ban, highlighting the importance of avoiding overfishing to ensure sustainable fisheries production. Therefore, it is believed that extending the fishing ban or adjusting fishing cessation regulations in the presence of guilds can facilitate the sustainable recovery of large yellow croaker resources in the East China Sea, as well as other fishery resources.

Previous reports indicate that the minimum body length and weight for the sexual maturation of female large yellow croakers are 220–240 mm and 200 g, respectively, and those for males are 200–220 mm and 150 g [36]. In the present study, the average body length of the sampled large yellow croaker was 141.73 mm, with an average weight of 48.32 g. Therefore, the caught large yellow croakers were smaller than the minimum body length and weight required for reaching sexual maturity. This uncontrolled fishing of immature large yellow croakers hinders the replenishment of mature individuals in the crucial spawning grounds of the East China Sea, thereby making it challenging for spawning seasons to regenerate the population. Thus, even under the background of large-scale stock enhancement, the recovery of large yellow croaker resources is still quite difficult. We also discovered that the gonadal development of some small-sized individuals reached the V-stage of sexual maturity prematurely. This indicates that the large yellow croaker has a tendency to reach sexual maturity earlier and transform into an R-strategy selector. In the study by Zhou et al. [30], utilizing the yield per recruit model, the authors calculated the F_0.1_ and F_max_. Their findings revealed that F_0.1_ < F_max_ < F_cur_, indicating that regardless of whether the F_0.1_ or F_max_ is used as the reference point for fisheries management, the nearshore large yellow croaker population in the East China Sea is in a state of overexploitation. Uncontrolled fishing is actually destroying the large yellow croaker fishery resources, which have just begun to recover. Therefore, the failure to recover the large yellow croaker fishery resources has further promoted the implementation of stock enhancement, and the impact of stock enhancement on the genetic diversity of wild fishery resources and the associated genetic risk has also attracted attention. Some previous studies on stock enhancement for other species have shown that stock enhancement could have potential genetic impacts on wild populations of the released species. For instance, the large-scale release of red sea bream in Kagoshima Bay, Japan, has resulted in genetic impacts on wild populations, ultimately leading to the loss of rare alleles through genetic drift in wild individuals [37], and potential negative genetic impacts could lead to the collapse of the wild resource and possibly make stock enhancement efforts counterproductive. There have been no previous studies on the genetic diversity of large yellow croaker individuals released for stock enhancement; thus, the impact of stock enhancement releases on the genetic diversity of the wild population of the large yellow croaker is unclear. It is necessary to control the fishing intensity and to pay special attention to the long-term genetic effects of stock enhancement to better maintain the wild population of the large yellow croaker.

### 4.3. Suggestions Regarding the Management of the Large Yellow Croaker Fisheries

The large yellow croaker stock enhancement program aimed at restoring the population involves releasing hatchery juvenile fishes into the wild to restore the seriously depleted spawning population and allow it to provide regular, substantial yields again [25]. Overfishing is widely recognized as one of the main causes of fishery resource degradation worldwide, and it can lead to a direct reduction in the resource through removing the target species from the water. Therefore, the recovery and sustainable utilization of resources can only be achieved by maintaining a dynamic balance between fishing and releasing.

For the past two decades, the implementation of the fishing ban policy by the Chinese government has garnered widespread support from fishermen and has yielded positive ecological, economic, and social benefits. However, there are still numerous issues that need to be addressed [3]. In the present study, we showed that due to the lack of timely, effective, or adequate management, heavy fishing pressure, especially transition fishing for the early-life-stage large yellow croaker in spawning and wintering grounds, is the main factor that has contributed to the decline of its resources and its inability to recover. During the fishery production process after the fishing ban period, the guild composed of large and small yellow croakers was the focus of indiscriminate intensive fishing, and many young large yellow croakers were caught, which readily causes the size cut-off effect, thereby affecting the reproduction, growth, and ecological role of the population [38]. Due to the migratory and feeding habits of the large yellow croaker, which cover vast geographical areas, the protection and management processes of the fishery resources of this species are complex and require strong management and enforcement. If fishing activities are not scientifically and adequately regulated, it will be difficult to achieve ideal results when releasing migratory fish such as the large yellow croaker back into the wild.

The concept of a guild can be used to answer important fisheries management questions, such as how community composition varies with habitat [16], how fishing development affects food web stability [39], and how fishing induces changes in ecosystem processes [40]. We consider that given the formation of the guild of large and small yellow croakers, resource protection for the large yellow croaker must be coordinated with the protection of small yellow croaker resources. An important example is the establishment of the National Aquatic Germplasm Resource Protection Area for Ribbon Fish in the East China Sea by Zhejiang Province in 2008. Since 2013, fishing has been prohibited in the core area of the protection area, with a fishing ban lasting approximately 1 month longer than the East China Sea fishing ban period. These conservation measures have led to increases in the biomasses of both the large yellow croaker and ribbon fish (*Trichiurus lepturus*) [41]. Currently, the existing single protection measures for the large yellow croaker are not sufficient. Instead, we advocate for the development of a collaborative conservation strategy for the marine region known as the “spawning ground, nursing ground, overwintering ground, and migration route”, where the guild consisting of the large yellow croaker and the small yellow croaker can thrive.

Given the aforementioned issues and the need to ensure the recovery of wild large yellow croaker resources and the effectiveness of the fishing ban in the spawning season, we provide the following recommendations: (1) The fisheries authorities should regulate the number and total power of fishing boats, adjust the marine fisheries structure, allocate a portion of the funds for large yellow croaker stock enhancement toward labor transition and placement, and gradually reduce the intensity of marine fishing each year. (2) By treating a guild as a management unit, the catch of the small yellow croaker can be used to estimate the utilization level of the large yellow croaker fishery resources. (3) Gradually extend the fishing ban period for the “spawning ground, nursing ground, overwintering ground, and migration route” used by the guild, transition the ban on trawl, sail-style net, and gillnet fishing to year-round prohibition, and only allow hook-and-line fishing; accelerate the implementation of the total allowable catch system (TAC) for major economic fish species; and establish net mesh size specifications to gradually restore the large yellow croaker stock resource. (4) Develop recreational fishing and support the construction of marine farms. Develop offshore marine aquaculture and strengthen research into scientific utilization to promote ecological restoration while ensuring economic development.

## 5. Conclusions

In conclusion, this study demonstrated that when the released and enhanced large yellow croaker grew to an average body length of 141.73 ± 14.61 mm and an average body weight of 49.45 ± 17.77 g in the wild, large yellow croaker individuals of this size exhibited a life history strategy of cohabitation with the small yellow croaker, and the two formed a guild. Based on this phenomenon, fishing targeting the small yellow croaker after the end of the fishing ban would impose significant fishing pressure on the large yellow croaker population, ultimately leading to the failure of China’s long-standing large yellow croaker stock enhancement activities to achieve their desired effects.

## Figures and Tables

**Table 1:** Proportions of large and small yellow croakers caught in different sites in the East China Sea.

Province and City	Sampling Site	*N.* of Samples	*N.* of *L. crocea*	*N.* of *L. polyactis*	Proportion of *L. crocea* (%)	Proportion of *L. crocea* in Different Province (%)	Total Percentage
Jiangsu	Lvsiport	326	37	299	11.35%	15.77 ± 9.15%	21.44 ± 9.35%
	Yangkou port	232	59	173	25.43%		
	Doulong port	550	128	422	23.27%		
	Yanwei port	220	12	208	5.45%		
	Lianyungang	356	22	334	6.18%		
Shanghai	Luchao port	340	78	262	22.94%		
Zhejiang	Longwan	696	199	497	28.59%	25.91 ± 7.53%	
	Taizhou port	986	336	650	34.08%		
	Shipu port	1408	349	1059	24.79%		
	Shengjiameng	1460	236	1224	16.16%		
Fujian	Chidian town	240	65	175	27.08%	24.69 ± 8.78%	
	Pinhai town	50	8	42	16.00%		
	Mawei district	862	128	734	14.85%		
	Luoyuan bay	164	53	111	32.32%		
	Sansha bay	440	146	294	33.18%		

**Table 2:** Regional variations in body length and body mass composition for the large yellow croaker in the East China Sea.

Province	Specie	Body Length/mm			Body Mass/g		
		Range	Dominant Group	Mean ± SD	Range	Dominant Group	Mean ± SD
Jiangsu	*L. crocea*	118–187	130–160	143.17 ± 14.04	12.7–116.2	30–60	46.65 ± 15.33
	*L. polyactis*	125–176	120–150	140.91 ± 10.17	33.54–89.22	30–60	47.73 ± 10.46
Zhejiang	*L. crocea*	104–166	130–160	140.5 ± 13.451	16.78–75.3	30–60	41.17 ± 11.82
	*L. polyactis*	98–160	100–130	136.31 ± 14.24	15.32–55.82	20–50	37.44 ± 10.69
Fujian	*L. crocea*	99–183	130–160	141.79 ± 16.57	22.06–135.08	40–80	61.91 ± 18.89
	*L. polyactis*	111–169	140–170	138.14 ± 16.93	24.58–80.1	20–70	54.76 ± 11.54

**Table 3:** The δ^13^C (‰) and δ^15^N (‰) values for the two fish species in the Zhejiang sea area.

Region	Species	Number	δ^13^C		δ^15^N		Trophic Level
			Range	Mean ± SD	Range	Mean ± SD	
Zhoushan	*L. crocea*	10	−16.80~−15.88	−16.22 ± 0.51	10.52~11.94	10.84 ± 0.17	4.16 ± 0.05
	*L. polyactis*	10	−17.32~−15.30	−16.31 ± 1.01	10.69~11.56	10.79 ± 0.11	4.15 ± 0.03
Taizhou	*L. crocea*	10	−17.22~−16.95	−17.11 ± 0.14	10.27~10.56	10.39 ± 0.15	4.03 ± 0.04
	*L. polyactis*	10	−18.64~−16.35	−18.06 ± 1.5	9.95~12.04	10.84 ± 1.07	4.16 ± 0.32

**Table 4:** Analysis of alpha diversity indices obtained for intestinal microbiota in the two species in different sea areas.

Groups	Numbers of Sequences	ACE	Chao1	Shannon	Simpson
LZS	91,217	500.19 ± 347.17	473.91 ± 320.92	2.4 ± 0.45	0.19 ± 0.04
SZS	94,712	549.4 ± 363.46	523.16 ± 329.18	2.34 ± 0.32	0.21 ± 0.01
LTZ	83,392	656.37 ± 332.40	615.2 ± 298.06	2.38 ± 0.45	0.2 ± 0.03
STZ	70,322	810.32 ± 16.19	763.01 ± 16.53	2.5 ± 0.05	0.21 ± 0.01

## Data Availability

The data presented in this study are available on request from the corresponding author.
